# A Preliminary Experience of Implementing Deep-Learning Based Auto-Segmentation in Head and Neck Cancer: A Study on Real-World Clinical Cases

**DOI:** 10.3389/fonc.2021.638197

**Published:** 2021-05-05

**Authors:** Yang Zhong, Yanju Yang, Yingtao Fang, Jiazhou Wang, Weigang Hu

**Affiliations:** ^1^ Department of Radiation Oncology, Fudan University Shanghai Cancer Center, Shanghai, China; ^2^ Department of Oncology, Shanghai Medical College, Fudan University, Shanghai, China; ^3^ Shanghai Key Laboratory of Radiation Oncology, Shanghai, China

**Keywords:** clinical evaluation, head and neck cancer, organs at risk, deep learning, auto segmentation

## Abstract

**Purpose:**

While artificial intelligence has shown great promise in organs-at-risk (OARs) auto segmentation for head and neck cancer (HNC) radiotherapy, to reach the level of clinical acceptance of this technology in real-world routine practice is still a challenge. The purpose of this study was to validate a U-net-based full convolutional neural network (CNN) for the automatic delineation of OARs of HNC, focusing on clinical implementation and evaluation.

**Methods:**

In the first phase, the CNN was trained on 364 clinical HNC patients’ CT images with annotated contouring from routine clinical cases by different oncologists. The automated delineation accuracy was quantified using the Dice similarity coefficient (DSC) and 95% Hausdorff distance (HD). To assess efficiency, the time required to edit the auto-contours to a clinically acceptable standard was evaluated by a questionnaire. For subjective evaluation, expert oncologists (more than 10 years’ experience) were randomly presented with automated delineations or manual contours of 15 OARs for 30 patient cases. In the second phase, the network was retrained with an additional 300 patients, which were generated by pre-trained CNN and edited by oncologists until to meet clinical acceptance.

**Results:**

Based on DSC, the CNN performed best for the spinal cord, brainstem, temporal lobe, eyes, optic nerve, parotid glands and larynx (DSC >0.7). Higher conformity for the OARs delineation was achieved by retraining our architecture, largest DSC improvement on oral cavity (0.53 to 0.93). Compared with the manual delineation time, after using auto-contouring, this duration was significantly shortened from hours to minutes. In the subjective evaluation, two observes showed an apparent inclination on automatic OARs contouring, even for relatively low DSC values. Most of the automated OARs segmentation can reach the clinical acceptance level compared to manual delineations.

**Conclusions:**

After retraining, the CNN developed for OARs automated delineation in HNC was proved to be more robust, efficiency and consistency in clinical practice. Deep learning-based auto-segmentation shows great potential to alleviate the labor-intensive contouring of OAR for radiotherapy treatment planning.

## Introduction

Radiation therapy represents one of the primary treatment modalities used in the management of head and neck cancer (HNC). Advanced radiotherapy techniques, such as intensity-modulated radiotherapy (IMRT), stereotactic body radiotherapy (SBRT), and volumetric-modulated arc therapy (VMAT) facilitate high conformal radiation doses to the tumor target while sparing of normal tissue to reduce the radiation toxicity (1). One of the most challenging steps in radiotherapy treatment planning is accurate delineation of the target volume and the adjacent organs at risk (OARs). The drawbacks of manual delineation of the OARs of HNC is that it is extremely time-consuming, labor-intensive and subject to the variability of the radiation oncologists’ anatomical knowledge (2–5).

Segmentation of HNC CT images accurately and automatically is a challenging due to the following three reasons: (1) The complexity and variability of the underlying anatomies are high; (2) Many anatomical structures involved in segmentation are relatively small in terms of their volumes; (3) The contrast of soft tissues is poor in the CT images. One of the common methods for automatic OARs segmentation is atlas-based auto-segmentation (ABAS) (6–8). For patients with HNC, atlas-based models may achieve acceptable image delineation for OARs (9, 10), but a clinical quality segmentation requires a tremendous atlas database under the assumption of perfect atlas selection (4, 11–14). Additional modification of contours is required, with a long execution time after ABAS, which does not reduce time in the segmentation workflow (15, 16). Lately, the focus has turned to deep learning (DL)-based methods due to their great success in medical image segmentation (12, 17–21). The major evident advantage of DL-based autosegmentation is that it can systematically learn the adequate features, which was never possible with the naked eye for segmentation, from a large amount of a given training database. Then, the same features can be searched automatically in a validation set (22).

Although, DL-based methods have achieved impressive results in OARs auto segmentation for HNC radiotherapy (18, 23–25), prospective clinical application of this technology remains stymied by two key challenges. First, the majority of these studies trained on selective training sets or databases from single open-access resource (26, 27). These datasets are limited in diversity. Therefore, to improve the performance of architecture, the network should rely on a much larger range of annotated dataset, which covers diverse real-world routine clinical cases. Second, it is extremely difficult to compare the segmentation performance between these state-of-the-art techniques directly, because they do not, in general, provide detailed statistical descriptions (e.g., image acquisition setups, image properties, manual delineation guidelines and patient cohorts) of the corresponding gold-standard. Furthermore, different performance metrics are often used in these studies for different OARs (22). As an emerging clinically relevant tool, therefore, adequate assessment methods that relate more directly to clinical judgment of contours are required.

Hence, in this study, not only the performance metric but also a clinical evaluation was introduced to evaluate the performance of convolutional neural networks (CNNs). Combining these two approaches can provide a more comprehensive approach for the evaluation of the clinical acceptance level of automatic contouring. Moreover, a larger range of annotation datasets relevant to real-world clinical routine cases were included in our architecture training. To improve the performance of the auto-segmentation model, a two-phase training was conducted in our study. The retraining data came from the first phase, which were edited by experienced oncologists until they believed them to be clinically acceptable.

## Materials and Methods

### Study Design

**Figure 1:**
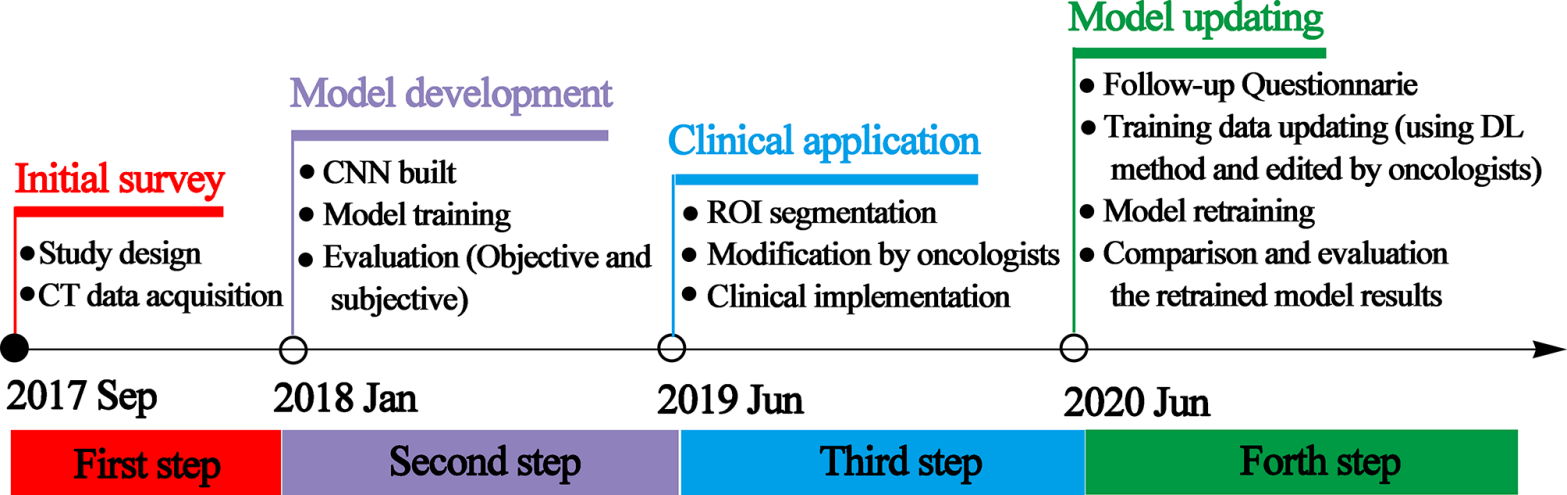
Schematic of the study design representing the timeline and the details of every step.

The workflow of this study can be divided into four steps: initial survey, model development, clinical implementation, and model updating. The time and details of every step are presented in **Figure 1**.

### CNN and Patients

A U-net similar network was implemented in this study. The details of the network architecture were presented in our previous study (28). The number of filters for each convolutional layer was 64, 64, 128, 256, and 512, respectively, with the feature map size reduced by half after the max-pooling layer. All convolutional layers applied 3×3 kernel. A 5 channels input tensor were input into the network. The output data was the 15 contouring of OARs. The model was implemented in Keras, and the loss function used in training process was dice index. The network was trained for 200 epochs with a learning rate of 1e-4. The optimizer is RMSprop. It took about 3 - 5 days to complete the whole training procedure.

Images from 364 HNC patients were included. All patients were treated with primary curative radiotherapy, with or without systemic treatment, between January 2015 and September 2017 at the Fudan University Shanghai Cancer Center (FUSCC). According to the conventional clinical protocol, each patient underwent a contrast-enhanced planning CT scan in the supine position with a custom thermoplastic mask for immobilization. The CT images were made on a multidetector-row spiral CT scanner Philips Brilliance Big Bore (Philips Healthcare, Cleveland, OH). The acquisition parameters were: 350 mA tube current, 120 kVp tube voltage, 0.92 × 0.92 mm pixel size, 5 mm thickness, 512 × 512 matrix. All of the training datasets were delineated by oncologists in our center. A total of 15 OARs were contoured, including the spinal cord, lens, brainstem, parotid gland, temporal lobe, oral cavity, larynx, eyeball, optic nerve and optic chiasm.

### Quantitative and Subjective Evaluation

In this study, the results evaluation and analysis were mainly divided into two parts: quantitative evaluation and subjective evaluation. For quantitative evaluation, the similarity between the automatic and manual contours in clinical practice is often assessed by calculating the “Distance”. The geometric accuracy of each approach was evaluated by comparing the difference between the automated segmentation and the manual segmentation using two metrics: the Dice similarity coefficient (DSC) (29) and the Hausdorff distance (HD) (30). Segmentation accuracy was quantified using both of them by measuring the degree of mismatch between the automatically generated (A) and manual contours (B). The DSC is calculated as $DSC=2*\frac{\left|A\cap^{}B\right|}{\left|A\right|+\left|B\right|}$which quantifies the overlap between contours A and B. It ranges from 0, indicating no spatial overlap between the two segmentations, to 1, indicating a complete overlap.

The HD is the greatest of all the distances from a point in A to the closest point in B. Smaller values usually represent better segmentation accuracy. HD95 considers the 95^th^-percentile of the closest point distances instead of their maximum:

${\text{HD}}_{95\text{th}}\left(X,\;Y\right)=\max\left({\text{h}}_{95}\left(\text{X},\text{Y}\right),{\text{h}}_{95}\left(\text{Y},\text{ X}\right)\right)$

Where ${\text{h}}_{95}\left(\text{X},\text{ Y}\right)={\text{K}}_{\text{x}\in \text{X}}^{95\text{th}}\underset{\text{y}\in \text{Y}}{\min}\left\{|\text{x}-\text{y}|\right\},$and ${\text{K}}_{\text{x}\in \text{X}}^{95\text{th}}$ is the kth ranked minimum Euclidean distance with K/N_x_ = 95%.

A subjective evaluation of the contouring methods was carried out to further analyze the deficiency of automated contouring by the model and its clinical usability. A nasopharyngeal carcinoma (NPC) panel (2 or 3 oncologists with more than 10 years’ experience) were invited to grade the results of contouring of 30 patients in the predictive data. A total of 900 organ contours (15 ROIs for each patient) were divided into 60 random combination queues. The observers were blinded to the origin of the contours in each session. During the evaluation process, the oncologist does not know whether the outline of the current evaluation was drawn automatically or manually. The evaluation was completely determined by the actual contouring effect. In this way, doctors’ subjective bias can be avoided as much as possible.

The evaluation method mainly includes two aspects: On the one hand, the oncologists make a comprehensive evaluation of the position, contour and edge details of each organ at risk. There are four evaluation levels for clinical use. Would you:

- ‘‘Require it to be corrected; there are large, obvious errors”,
- ‘‘Require it to be corrected; there are minor errors”,
- ‘‘Accept it as it is; but it needs a small amount of editing”,
- ‘‘Accept it as it is; the contour is very precise”.

On the other hand, two contours were blindly presented with random slices: “which contour do you prefer?” There are five scales:

1. Strong tendency to manual
2. More inclined to manual
3. No tendency
4. More inclined to auto
5. Strong inclination to auto

Meanwhile, a follow-up questionnaire was conducted, which was test piloted among 17 experienced oncologists from different institutions. More details about this questionnaire can be found in Appendix I. The results are presented in part 3.1.

### Model Updating

The DL-based autosegmentation is strongly based on reference OAR delineations in the given image database. With the help of the auto-contouring method, physicians may have more time to focus on the details, delineations, and modifications. These improved delineations can be used for model updating. Therefore, another three hundred patients (300) were collected and used for model retraining without updating model hyperparameters. The 15 OARs were automatically generated by the pre-training CNN network. All delineations were verified and approved with or without modification by the oncologists to ensure their clinical validity.

## Results

### Results of the Questionnaire

**Figure 2:**
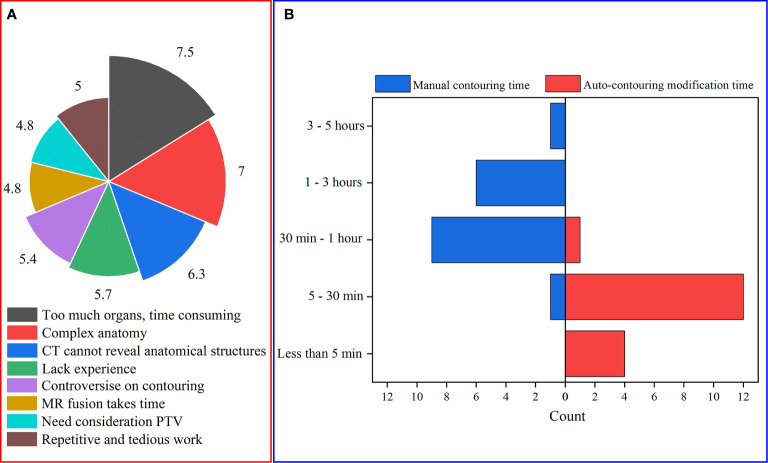
**(A)** The main obstacles of HNC OAR delineation (to present the questionnaire data, for each issue every item was given corresponding values according to the ranking. Therefore, the average score of every item can be obtained); **(B)** The time consumed for manual contouring and auto-contouring modification.

**Figure 2A** shows that too many organs and the complex anatomical structure of the OARs are the two main obstacles for HNC OAR delineation. The time for manual delineation and modification of auto-contours are shown in **Figure 2B**. It can be seen that for NPC, the manual time of contouring varies from more than 3 hours to less than 30 minutes for different oncologists without the assistance of a DL-based method. Compared with the manual delineation time of one HNC patient, after using auto-contouring this duration was shortened from hours to minutes. The minimum time of contouring can be less than 5 minutes. From this, we concluded that the DL-based method has great potential to reduce the delineation time required to produce acceptable contours for oncologists.

### Qualitative Evaluation

To investigate the accuracy, the HD and DSC values of the OAR segmentation for the two training times are summarized in **Table 1**. The network performed well in the first training for the spinal cord, brainstem, temporal lobe, eyes, optic nerve, parotid glands and larynx [with a mean DSC >0.7 as the “good” criteria (2)], especially with the best contour similarity of the left and right eye, reaching approximately 0.83. The mean DSC for all other structures was below 0.6. The corresponding evaluation parameters for HD are listed in the second column of **Table 1**.

**Table 1:** The DSC and HD values of 2 evaluation parameters for the 15 OARs segmentation.

	HD*(mm)	DSC*(%)	HD**(mm)	DSC**(%)
**Spinal cord**	9.03 ± 0.11	0.79 ± 0.04	6.36 ± 2.96	0.87 ± 0.06
**Brain stem**	7.91 ± 1.64	0.79 ± 0.01	9.05 ± 3.05	0.80 ± 0.06
**Lobe-R**	27.47 ± 6.99	0.72 ± 0.06	14.53 ± 6.9	0.88 ± 0.073
**Lobe-L**	23.26 ± 6.00	0.73 ± 0.06	14.28 ± 8.56	0.87 ± 0.09
**Eye-R**	6.13 ± 1.73	0.82 ± 0.03	3.86 ± 1.66	0.93 ± 0.04
**Eye-L**	5.62 ± 0.93	0.83 ± 0.02	3.23 ± 1.94	0.93 ± 0.05
**Lens-R**	5.19 ± 0.34	0.51 ± 0.09	2.75 ± 1.81	0.78 ± 0.16
**Lens-L**	5.06 ± 0.91	0.56 ± 0.10	3.50 ± 3.30	0.71 ± 0.19
**Oral cavity**	23.04 ± 6.55	0.53 ± 0.12	8.51 ± 5.54	0.93 ± 0.07
**Optic nerve-R**	10.55 ± 2.12	0.44 ± 0.06	6.72 ± 3.6	0.65 ± 0.21
**Optic nerve-L**	7.82 ± 1.94	0.51 ± 0.10	6.50 ± 8.9	0.69 ± 0.18
**Parotid-R**	12.73 ± 3.40	0.79 ± 0.05	7.04 ± 4.56	0.92 ± 0.07
**Parotid-L**	14.06 ± 4.60	0.79 ± 0.04	7.3 ± 4.5	0.85 ± 0.13
**Larynx**	13.01 ± 1.73	0.72 ± 0.04	8.93 ± 3.55	0.84 ± 0.08
**Chiasm**	9.95 ± 2.63	0.46 ± 0.11		

*the results of model trainings; **the results of model updating.

For the retraining model, the performance of the network was improved significantly for the OARs in HNC patients. The largest DSC score increase was 0.4 for the oral cavity (14.53 mm for 95% HD). For clearly visible boundaries organs, the mean DSC scores increased from 0.79 to 0.87 for the spinal cord, from 0.72 to 0.84 for the larynx, from 0.82 to 0.93 for the left eye, from 0.83 to 0.93 for the right eye, from 0.79 to 0.92 for the right parotid and from 0.79 to 0.85 for the left parotid. Similar observations could also be acquired for 95% HD in the fourth column of **Table 1**. For small organs, the retraining process could significantly improve the performance of delineation (such as the optic nerve, lens and lobe). The average DSC scores improved to approximately 0.2, and the highest values appeared for the right lens (0.27). However, there was no improvement for the brainstem, and the corresponding HD parameter decreased by 1.14 mm. Moreover, the feature of the chiasm could not be delineated by our CNN net after the training data updating.

### Subjective Evaluation

**Figure 3:**
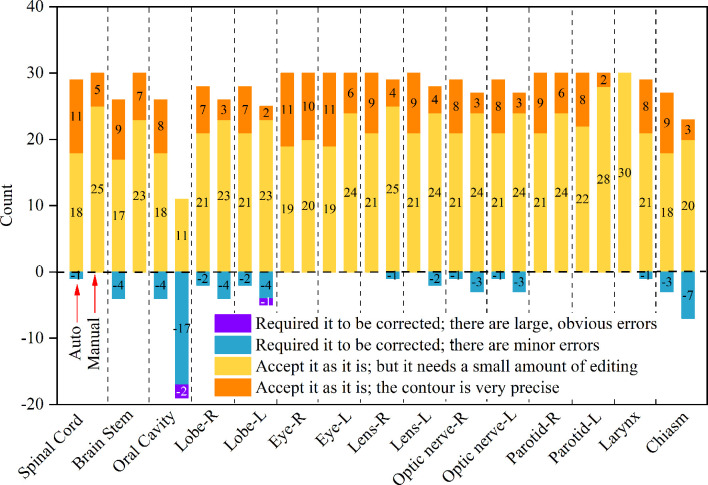
The evaluation of the OARs for the network performance by clinicians. The left bar is the count number by the auto segmentation method and the right bar is manual.

The results obtained by the physicians after the grade assessments are shown in **Figure 3**. From the graph, it can be seen that whether it is auto or manual contouring, most of the contouring organs are graded “very satisfied” or “minor modification but still can be used for clinical applications”. A huge deviation only appears for the oral cavity in the group of manual contours.

**Figure 4:**
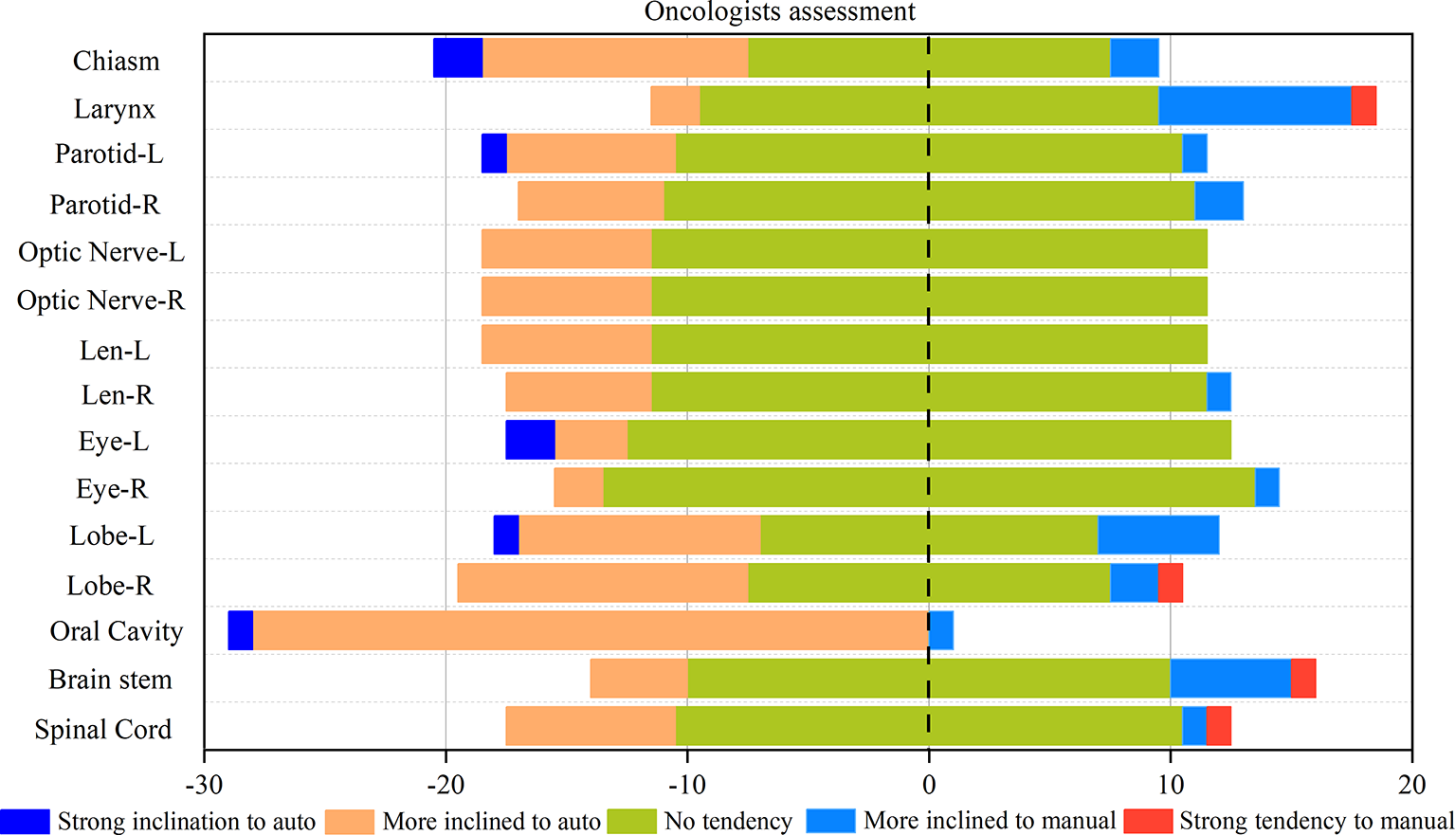
Rates of correct and incorrect classification of contours as human or automatically generated.

**Figure 4** shows the statistical map of the selection tendency between the results of the auto and manual contouring. Except for the oral cavity, the majority of choices are the “no tendency” in the blind selection. This means that for oncologists, for the majority of cases the results of auto-contouring and manual contouring are pretty close. In most cases, the automatic delineations had more “strong inclination” ratings than the manual ones. Even in the small and unclear organs with high difficulty in delineation, such as the left and right lens and optic nerves, automatic delineation also shows a good performance. However, for the larynx and brain stem, there is more of an inclination toward manual-contouring cases.

## Discussion

For measuring the geometrical agreement with the corresponding ground truth, the DSC is usually used. This parameter has been proven to be useful for larger-volume structures. For smaller organs, the performance was poor (31). This conclusion is consistent with the findings of our study. As seen, DSC is a relative volumetric measurement. The bigger the organ, the smaller the relative error becomes (31). Again, it is important to mention that the results of the DL-based methods, human performance, and commercial software are not a direct comparison obtained on the same database. Therefore, the results of DSC can only be part of the assessment of the performance of the DL methods. To further evaluate the performance of the CNN, apart from the volumetric overlap of two segmentation masks, automatic delineation results should have to also be evaluated from a clinical point of view. The effect of efficient autosegmentation on the clinical workflow may still be clinically relevant.

**Figure 5:**
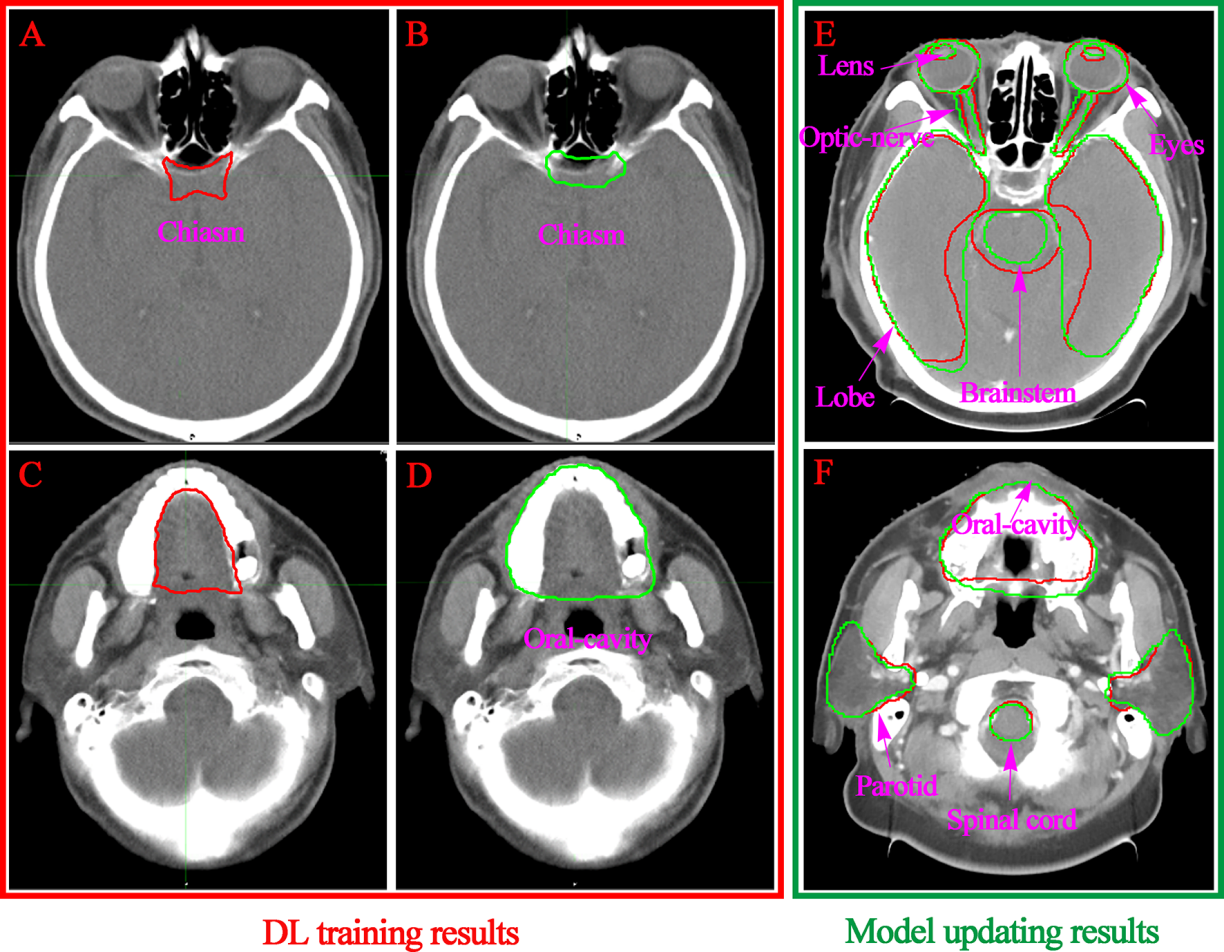
Visualization of the convolution neural network-based segmentation results of the chiasm **(A, B)** and oral cavity **(C, D)**. The DL performance of the OARs with model updating are listed in the right column **(E, F)**. The ground truth segmentations are depicted in red, and the auto segmentations are depicted in green.

A subjective assessment was performed. The comparison between the contours of the manual and DL (**Figure 3**) suggests that the performance of auto-contouring for most of the OARs were acceptable by oncologists and decreased the intra- and inter observer variability except for the oral cavity and chiasm. For these two OARs, the oncologist’s perspective is that part of the manual-contouring lacks inclusion of the teeth, and some of them include part of the structure of the larynx as shown in **Figures 5C, D**. For further evaluation of the quantitative assessment between auto and manual-contouring, the results of the classification of contours as human or automatically generated are shown in **Figure 4**. This suggests that the auto-contouring outperforms the manual-contouring (excluding the larynx and brainstem). The delineation problem of the brain stem is mainly reflected in the scope of the contouring; namely, there are a few more layers in the upper and lower boundaries. For the larynx, the physicians gave the opinion that the larynx is not included bone in some cases. From the comparison of the images, it can also be seen that the greatest delineation difference is the optic chiasm. The manually delineated optic chiasma (**Figure 5A**) presents a fuzzy shape, while the automatically delineated optic chiasma (**Figure 5B**) is relatively obvious in shape.

To improve the performance of the DL-based model, a number of automated and semiautomated methods have been proposed to address this problem. Meanwhile, the DL-based autosegmentation performance is also strongly based on the quality and representativeness of the training data. In this study, therefore, we collected an additional 300 CT images, which were generated by the CNN model and modified by oncologists in our center to ensure their clinical validity. The retraining model DSC values of the OARs are shown in **Table 1**. Except for the optic nerve and chiasm, all other OAR DSCs produced in our research were larger than 0.7, which is viewed as acceptable in practice (32). For all OARs, the retraining results outperformed the first-time training results. The largest DSC improved for the oral cavity from 0.53 to 0.93. The reason why there was a better performance of the retrained model can be explained as follows: As a supervised technique, CNNs considerably relies on annotated OAR delineations in the given image database. It has been demonstrated that enriching the training dataset may contribute to more accurate and acceptable segmentation. If the training database contains low quality or inconsistent images, it cannot represent the actual manual delineations of the OAR. The underlying automated segmentation network will either fail to train or will produce inaccurate or inconsistent delineation.

**Figure 6:**
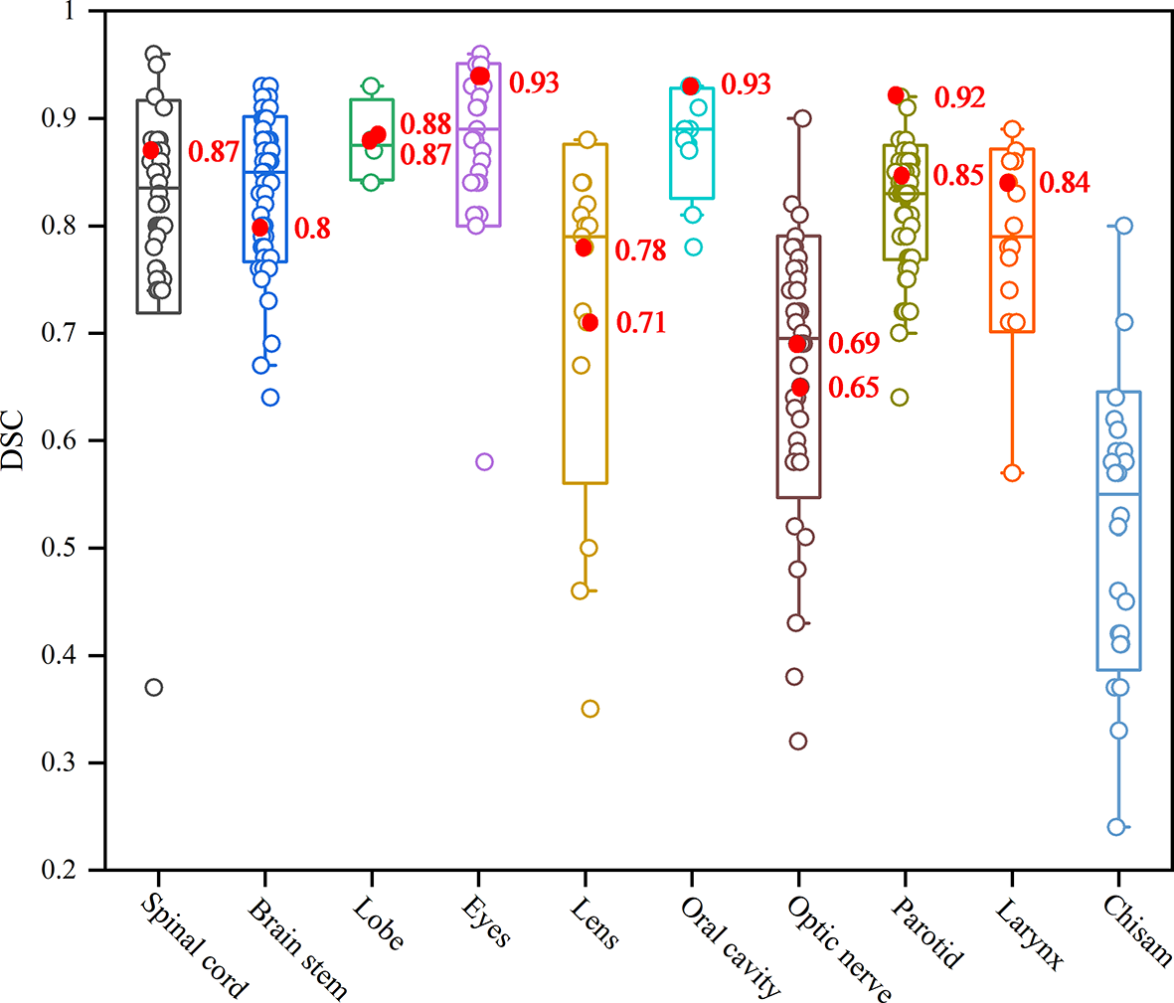
The box plot results of auto-segmentation of OARs in HNC reported in terms of the Dice coefficient (22). The red mark dots are the Dice coefficient in our second phase training results.

We summarized the studies previously reported on the topic of HNC OARs segmentations, comparing the published results with the proposed retrained model performance for individual OARs, as shown in **Figure 6**. It can be seen that the values of the DSC in our study outperformed the most current state-of-the-art nets for the spinal cord, lobe, eyes, oral cavity, parotid and larynx. Only the small and unclear boundaries organs, such as the lens and optic nerve in our study, had a lower DSC than the average results of the existing segmentation methods. For the chiasm, it is a pity that our net failed to segment it in the second phase of training. This shortcoming is the subject of our next research project.

Segmentation of an OAR strongly depends on its size, shape, clarity of boundaries, presence of pathologies, and overall visibility in the CT image modality (21). For the small tissues, such as the lens and optic nerve, it is difficult to identify the contour accurately even in the manual delineation process. Similar conclusions could be drawn from the previous research. A relatively low accuracy of DSC values [e g., 0.38 (31) and 0.39 (33)] were found because of the small size and unclear boundaries of the optic nerves. For large size and clear boundaries organs, such as the eyeball, brain stem, spinal cord, temporal lobe and oral cavity, it is quite difficult to directly see the differences in the same layers of the CT images (**Figures 5E, F**). We obtained segmentation results of larger than 0.87 DSC for these well-defined shapes and clear visibility OARs that were superior or comparable to the best performing automated segmentation. The position of the OARs in all predicted CT images can be accurately located, which indicates that the two-phase model training was successful.

A limitation of this study is that although the autosegmentation methods do decrease the required contouring time and the intra/inter observer variability, from the viewpoint of radiotherapy, both target volume and adjacent OARs delineation has direct clinical implications. The DL-based segmentation results should be assessed from the perspective of their dosimetric impact. This is because the relationship between the geometrical performance metrics and the dosimetric impact cannot be predicted. Even if the geometric differences are small, the impact on the final dose distribution may still be clinically relevant. Future studies should therefore focus on combining existing multiple geometric performance metrics with clinical dosimetric impact assessments for RT treatment.

## Conclusions

This study has two main new contributions or novelties summarized as follows. First, combining objective (performance metric) and subjective (clinical evaluation) assessment can provide a more comprehensive way to evaluate the clinical acceptance level of automatic contouring. Second, a two-phase training phase was conducted in our study to further improve the performance of the autosegmentation network. The model updating or retraining could significantly improve the performance of the delineation of the OARs in HNC patients and subsequent manual corrections that required considerably less time than direct manual delineation to produce acceptable contours in routine use.

## Data Availability Statement

The original contributions presented in the study are included in the article/**Supplementary Material**. Further inquiries can be directed to the corresponding authors.

## Author Contributions

Conception, design, and drafting the manuscript were performed by YZ, YY, JW, and WH. Data collection and interpreting were performed by YF. All authors contributed to the article and approved the submitted version.

## Funding

This work is supported the Shanghai Committee of Science and Technology Fund (19DZ1930902) and Xuhui District Artificial Intelligence Medical Hospital Cooperation Project (2020-009) and the Varian Research Grant (The deep learning based 3D dose prediction and automatic treatment planning).

## Conflict of Interest

The authors declare that the research was conducted in the absence of any commercial or financial relationships that could be construed as a potential conflict of interest.
